# From Microcirculation to Aging-Related Diseases: A Focus on Endothelial SIRT1

**DOI:** 10.3390/ph17111495

**Published:** 2024-11-07

**Authors:** Martin Law, Pei-Chun Wang, Zhong-Yan Zhou, Yu Wang

**Affiliations:** 1 Department of Pharmacology and Pharmacy, LKS Faculty of Medicine, The University of Hong Kong, Hong Kong SAR, China; martinlaw9770@gmail.com (M.L.);; 2 State Key Laboratory of Pharmaceutical Biotechnology, The University of Hong Kong, Hong Kong SAR, China; 3 Longhua Hospital, Shanghai University of Traditional Chinese Medicine, Shanghai 200032, China

**Keywords:** microcirculation, angiogenesis, artery aging, capillarization, endothelium

## Abstract

Silent information regulator sirtuin 1 (SIRT1) is an NAD+-dependent deacetylase with potent anti-arterial aging activities. Its protective function in aging-related diseases has been extensively studied. In the microcirculation, SIRT1 plays a crucial role in preventing microcirculatory endothelial senescence by suppressing inflammation and oxidative stress while promoting mitochondrial function and optimizing autophagy. It suppresses hypoxia-inducible factor-1α (HIF-1α)-mediated pathological angiogenesis while promoting healthy, physiological capillarization. As a result, SIRT1 protects against microvascular dysfunction, such as diabetic microangiopathy, while enhancing exercise-induced skeletal muscle capillarization and energy metabolism. In the brain, SIRT1 upregulates tight junction proteins and strengthens their interactions, thus maintaining the integrity of the blood−brain barrier. The present review summarizes recent findings on the regulation of microvascular function by SIRT1, the underlying mechanisms, and various approaches to modulate SIRT1 activity in microcirculation. The importance of SIRT1 as a molecular target in aging-related diseases, such as diabetic retinopathy and stroke, is underscored, along with the need for more clinical evidence to support SIRT1 modulation in the microcirculation.

## 1. Introduction

Microcirculation, which refers to the blood flow in vessels with a diameter of 100 μm or less, plays a key role in supplying nutrients and oxygen to the body. Microcirculation is characterized by a large surface area and slow blood velocity [1]. These vessels contribute to peripheral vascular resistance and control the trafficking of immune cells to tissues embedded deep within organs. Microcirculatory vessels are covered with microvascular endothelial cells (MECs), which secrete numerous factors, such as nitric oxide (NO), prostacyclin, and endothelin, to regulate vascular smooth muscle cell (VSMC) tone. During aging, gradual microvascular dysfunction (MD) due to chronic exposure to environmental and metabolic insults results in impaired organ function [2]. Age-associated micro-endothelial damage leads to capillary loss, impaired blood flow regulation, and diminished barrier function of substance exchange between the blood and organs or tissues [3]. Microvascular conditions are intricately linked to cardiovascular, neurodegenerative, and metabolic complications. In fact, MD often precedes age-related macrovascular conditions and predicts cardiovascular risk. For example, individuals with diabetic neuropathy and retinopathy, which are conditions characterized by MD, are more likely to exhibit coronary artery calcification [4]. In addition, MD may underlie various disease symptoms that are not currently well-diagnosed and treated. For example, coronary microcirculation dysfunction leads to stable angina without coronary artery obstruction [5]. There are currently no approved drugs specifically targeting MD. Thus, understanding the etiological mechanisms underlying MD is critical for the treatment and prevention of MD and aging-related diseases.

Silent information regulator 1 (SIRT1) is a nicotinamide adenine dinucleotide (NAD+) dependent deacetylase with broad physiological functions in proliferation, metabolism, and aging [6,7,8]. In humans, low SIRT1 levels during childhood are associated with MD later in life. Premature MD encompasses diminished vasodilation in response to NO production stimulated by acetylcholine delivered via iontophoresis, and reduced post-occlusive reactive hyperemia [9]. SIRT1 expressed in the endothelium elicits potent anti-arterial aging activities. Compared to non-obese humans, obese and elderly individuals exhibit lower micro-endothelial SIRT1 expression, reduced antioxidant proteins forkhead box O3 (FOXO3) and superoxide dismutase 2 (SOD2), and higher pro-oxidative mitochondrial-aging proteins p66Shc and arginase II, while incubation of human MECs with the SIRT1 activator SRT1720 rescues these changes [10]. Low SIRT1 levels correlate with increased fat deposition, vascular inflammation, LDL elevation, and obesity [11,12], while SIRT1 activation ameliorates insulin resistance, a reversible cause of vascular aging [13]. Furthermore, there is a positive association between serum SIRT1 and nitrate-mediated vasodilation and an inverse relationship between SIRT1 and noradrenaline, a vasopressor [14]. In aging-associated vascular dysfunction, endothelial SIRT1 levels are markedly diminished as a result of inflammatory and oxidative stress [15,16]. The loss of endothelial SIRT1 represents a crucial pathological factor and symptom of aging-related diseases. The present review summarizes recent evidence of endothelial SIRT1-mediated effects on microvascular structure and function in relation to aging-related diseases.

## 2. Regulation of Microcirculation by Endothelial SIRT1

Endothelial SIRT1 regulates a wide range of physiological functions in the microcirculation and plays a significant role in maintaining microcirculatory function under pathological conditions.

### 2.1. Regulation of Physiological Capillarization by SIRT1

Under physiological conditions, SIRT1 is required to maintain skeletal muscle capillarization and exercise capacity. Endothelial cell-specific deletion of SIRT1 (EC-SIRT1-KO) in mice results in a significant reduction in skeletal muscle capillary density, which was not observed in myocyte-specific SIRT1-knockout mice. Endothelial SIRT1 is also required for exercise-induced skeletal muscle capillarization and improvement in exercise capacity [17]. Exogenous supplementation of nicotinamide mononucleotide (NMN), the primary precursor of NAD+, in elderly mice activated SIRT1, restored skeletal muscle capillary density to levels typically observed in younger mice, and promoted exercise-induced capillarization in young mice. In humans, regular exercise increases plasma SIRT1 levels, correlating with improved mitochondrial function, NO bioavailability, and antioxidant capacity compared to a sedentary lifestyle [18,19]. Micro-endothelial SIRT1 also plays a crucial role in energy metabolism by regulating adipose tissue capillarization [20,21]. The capillary density of brown adipose tissue (BAT) is significantly reduced in EC-SIRT1-KO mice and increased following MEC-specific SIRT1 activation, but not in adipocyte-specific activation [22]. Moreover, lipid stimulation negatively impacts SIRT1 expression. Individuals with obesity exhibit markedly reduced microvascular SIRT1 expression and increased inflammation [10,23]. Olive oil consumption leads to diminished protein levels of SIRT1 in the mouse intestinal microvasculature [24]. Moreover, loss of SIRT1 following lipid stimulation is associated with mitochondrial dysfunction, inflammation, and decreased antioxidant protein expression [10], while activation of endothelial SIRT1 promotes angiogenesis, antioxidant protein expression, and mitochondrial function, and suppresses inflammation [10,21,24,25].

### 2.2. SIRT1 and Diabetic Microangiopathy

In diabetes, SIRT1 suppresses low-grade micro-endothelial inflammation induced by chronic high glucose (HG) exposure by downregulating inflammatory cytokine production and nuclear factor κB (NF-κB) activity while promoting NO bioavailability and endothelial function [10,26,27,28]. Activation of retinal micro-endothelial SIRT1 via fasting or fasting-mimicking treatment also improves retinal cholesterol export, decreases retinal cholesterol, and diabetes-induced retinal inflammation to improve retinal function [29]. While SIRT1 is traditionally thought to promote angiogenesis [30,31], upregulation of retinal micro-endothelial SIRT1 suppresses HG- and hypoxia-induced angiogenesis [27]. In diabetic kidney disease (DKD), endothelial SIRT1 dysfunction leads to peritubular capillary loss following kidney injury [32,33]. However, dysfunction of SIRT1 in DKD could also result in hypoxia-induced angiogenesis associated with kidney fibrosis [34,35]. In diabetic retinopathy, pathological angiogenesis under hypoxic conditions increases capillary permeability and retinal dysfunction [36,37]. In contrast, overexpression of SIRT1 suppresses diabetic angiogenesis to improve retinal function [27,38]. This shows that SIRT1 plays a dual role in micro-angiogenesis by suppressing pathological angiogenesis while promoting healthy angiogenesis.

### 2.3. SIRT1, Oxidative Stress and Mitochondrial Function

SIRT1 is also essential for combating the vicious cycle between oxidative stress and mitochondrial dysfunction [39]. By promoting mitochondrial biogenesis, structural integrity, antioxidant protein expression, and the destruction of dysfunctional ROS-producing mitochondria via mitophagy, SIRT1 ameliorates oxidative stress-induced myocardial hypertrophy, MEC injury, and apoptosis [35,40,41,42,43,44]. Downregulation of SIRT1 results in decreased autophagy and increased apoptosis, leading to the development of coronary microvascular dysfunction and diabetic retinopathy [45,46]. Furthermore, by regulating proteins involved in the mitochondrial respiratory chain, SIRT1 upregulates microvascular NO bioavailability while suppressing mitochondrial reactive oxygen species (mtROS) levels in ex vivo human micro vessels [10].

### 2.4. SIRT1 and Coronary Microvascular Function

Coronary microvascular dysfunction (CMD) is present in most patients with angina, adverse cardiac remodeling, and many other cardiovascular diseases [47,48]. SIRT1 deficiency contributes to CMD pathogenesis and cardiomyopathy by increasing microvascular oxidative stress [49]. The antioxidant effect of SIRT1 is also involved in its mechanism of protection against doxorubicin-induced cardiotoxicity [50,51,52], which is shown to significantly damage coronary microcirculation [53]. In addition, endothelial SIRT1 deficiency impairs angiogenesis, causing inflammation and platelet hyperactivation to exacerbate CMD and cardiomyopathy [54,55], while upregulation of SIRT1 ameliorates low myocardial capillary density and fibrosis [56].

### 2.5. SIRT1 and Brain Microvascular Function

Brain microvascular dysfunction (BMD) is a major cause of blood−brain barrier (BBB) hyperpermeability, substantially increasing the long-term risk of stroke [57,58]. Recent research implicates micro-endothelial SIRT1 in stroke and ischemia-induced brain damage. Aging-associated decline in brain microvascular SIRT1 expression plays a critical role in BBB permeability. SIRT1-KO mice exhibit increased BBB permeability, while SIRT1 overexpression or activation protects against senescence-associated endothelial hyperpermeability [59,60]. Post-ischemia, SIRT1 maintains the BBB by inhibiting inflammation, leukocyte adhesion, mtROS generation, and adverse remodeling [60,61,62,63]. Additionally, SIRT1 upregulates post-ischemic angiogenesis and mitophagy [64,65,66,67,68,69,70,71]. By regulating efficient energy metabolism, ROS, and inflammation during hypoxic-ischemic states, SIRT1 suppresses ischemia-induced brain microvascular endothelial cell (BMEC) apoptosis and promotes neuronal survival.

BMD is also closely linked to the pathogenesis of neurodegenerative diseases, substantially increasing the risk of vascular cognitive impairment, dementia, and Parkinson’s disease [72,73,74]. BBB dysfunction is also a major contributor to neurodegeneration [75], and improvement in hippocampal microvascular BBB function reduces chronic hypoperfusion-induced cognitive impairment [76]. The antioxidant and pro-angiogenic effects of SIRT1 are purported to be protective mechanisms against vascular cognitive impairment [77]. Of particular interest is the role of SIRT1 in suppressing neuronal amyloidogenesis, a key factor in the development of Alzheimer’s disease (AD). Inhibition of endothelial SIRT1 has been shown to promote amyloidogenic changes in BMECs following hypoxia-reperfusion injury, which could be reversed by overexpression or activation of SIRT1 [78,79]. Specifically, activation of SIRT1 shifts amyloid precursor protein processing toward the non-amyloidogenic pathway [79]. Donepezil, an acetylcholinesterase inhibitor used in the treatment of AD, is shown to promote angiogenesis, decrease BMEC permeability, and upregulate tight junction proteins via SIRT1-dependent pathways [69]. Current therapeutic options against cognitive decline are limited, and research on SIRT1 has provided a novel therapeutic target. Interestingly, chronic psychological stress is also associated with reduced SIRT1 levels, accelerated neuronal vascular senescence, and impaired angiogenesis [80]. Decreased levels of BDNF, a key protein implicated in neuropsychiatric pathologies including Alzheimer’s disease [81], correlate with lower levels of SIRT1 in depressed and schizophrenic individuals [82], potentially implicating SIRT1 in psychiatric disorders.

## 3. Mechanisms Underlying Regulation of Microcirculation by Endothelial SIRT1

**Figure 1:**
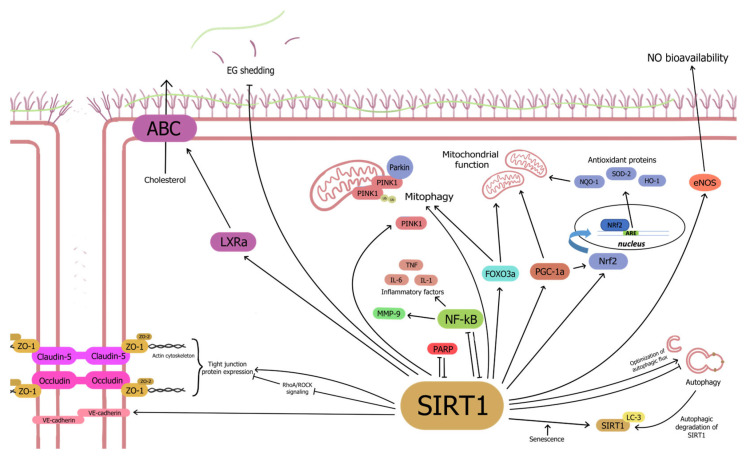
Endothelial silent information regulator 1 (SIRT1) exerts microcirculatory vasoprotective effects by upregulating antioxidant proteins, mitochondrial function, mitophagy, microcirculatory nitric oxide (NO) bioavailability, cholesterol export, and tight junction protein expression while suppressing inflammation and endothelial glycocalyx shedding, alongside optimizing autophagic flux.

Loss of microvascular endothelial function precedes impaired regulation of VSMC tone, angiogenesis, hemostasis, and thrombosis, leading to malperfusion and aging-related pathologies (Figure 1). The mechanisms underlying the effects of endothelial SIRT1 on microcirculation are discussed in this section.

### 3.1. SIRT1 Enhances Endothelium-Dependent Vasodilation in an eNOS-Dependent Manner

NO, produced by endothelial nitric oxide synthase (eNOS) in blood vessels, is the most crucial component of vascular reactivity. Oxidative stress-induced NOS decoupling is a common pathway for endothelial dysfunction and leads to further production of ROS [83]. eNOS upregulation by SIRT1 is one of the most well-documented sirtuin pathways. Deacetylation by SIRT1 directly increases eNOS activity and NO bioavailability [84]. In addition, SIRT1 indirectly upregulates eNOS via KLF2, FOXO1/FOXO3a [85,86], and cellular protection against ROS. There is also thought to be a positive-feedback loop between SIRT1 and eNOS, as inhibition of NOS also reduces the expression of SIRT1 [87], while NO-induced SIRT1 activation increases PGC-1α and improves mitochondrial function [88]. eNOS is required for the benefits of SIRT1 in endothelial function. SIRT1 activation by nicotinamide riboside promotes intestinal microcirculatory perfusion in necrotizing enterocolitis, which was not observed in eNOS-knockout mice, resulting in endothelial dysfunction due to limited NO production [89]. SIRT1 inhibition downregulates eNOS expression, whereas activation of SIRT1/eNOS signaling improves blood flow recovery following hindlimb ischemia [90]. GLP-1 agonists and DPP-4 inhibitors have also been shown to promote eNOS function, MEC survival, and proliferation via SIRT1 activation [91,92].

SIRT1 also enhances eNOS-independent vasodilation by regulating VSMC potassium channels to induce endothelium-dependent hyperpolarization (EDH)-mediated relaxation. In aged, spontaneously hypertensive rats, reduced SIRT1 is associated with loss of small conductance calcium-activated potassium channels (SKCa) and sodium-potassium ATPase (Na-K-ATPase), leading to impaired EDH-mediated vasodilation, effects that were reproducible upon SIRT1 inhibition via EX-527 [93]. EDH-type responses are enhanced in females, likely due to 17β-estradiol promoting the activity of intermediate conductance calcium-activated potassium channels (IKCa) [94]. Ovariectomized rats also exhibit diminished SKca activity [95]. Additionally, SIRT1 expression is upregulated by 17β-estradiol to prevent senescence and ED in ovariectomized animals [96], which may be a mechanism underlying sex differences in atherosclerotic cardiovascular disease [97].

### 3.2. Anti-Senescence Activity of Endothelial SIRT1

Cellular senescence, originally defined as the irreversible loss of proliferative potential in somatic cells, is a major factor in vascular dysfunction [39]. However, senescence is also characterized by distinct phenotypic changes in cellular morphology and function. While remaining metabolically active, they produce a vastly different secretome than non-senescent cells, also known as senescence-associated secretory phenotype (SASP) [98]. Accumulation of SASP-expressing endothelial cells during aging leads to MD. By suppressing senescence, endothelial SIRT1 maintains microvascular function and represents a major therapeutic target for tackling aging-related diseases [99].

#### 3.2.1. SIRT1-NF-κB Interaction Dictates Microvascular Function and Inflammation

NF-κB is a crucial transcriptional regulator of inflammation that is involved in upregulating a wide range of proinflammatory factors, including interleukin (IL)-18, IL-6, and tumor necrosis factor α (TNFα). SIRT1 targets the p65/relA subunit of NF-κB [100]. Deacetylation of this site inhibits nuclear translocation and promotes ubiquitin-proteasome-mediated degradation of NF-κB [101,102]. Microcirculatory inhibition of SIRT1 both directly and indirectly increases the activation of NF-κB, leading to ED, which can be reversed by SIRT1 overexpression or activation [23,24,62,69,103]. Additionally, deletion of toll-like receptor 4, an activator of NF-κB, could attenuate lipid-induced downregulation of SIRT1 expression [24]. In diabetic retinal microvascular endothelial cells (RMECs), NF-κB overactivation upregulates MMP-9, leading to mitochondrial damage, cell death, and subsequent retinopathy [104]. Inflammatory NF-κB overactivation also causes microvascular dysfunction by accelerating the shedding of microvascular endothelial glycocalyx [105], a mechanoresponsive factor in regulating NO-mediated vasodilation [106], which could be prevented by SIRT1 activation. Endothelial SIRT1-deficiency leads to the release of the syndecan-4 ectodomain, producing a profibrogenic signal to nearby endothelial cells, inhibiting eNOS activity and angiogenesis [106,107,108]. Conversely, NF-κB indirectly suppresses SIRT1 activity through its downstream factors. Exposure of intestinal MECs to TNFα, both a target and activator of NF-κB, has been shown to decrease SIRT1 activity associated with reduced NO production and ED [89].

#### 3.2.2. Senescence, Autophagy and SIRT1

SIRT1 protects cells from ischemia and inflammation by optimizing autophagy, a delicate process involving selective degradation and recycling of intracellular components [8]. In streptozotocin-induced diabetic retinopathy, upregulation of SIRT1 by stachydrine increases autophagy to reduce ROS and inflammation in RMECs [46]. Similarly, sponging of miR-200c-3p by lncRNA MALAT1 upregulated SIRT1 following BMEC Oxygen-Glucose Deprivation/Reperfusion (OGD/R), promoting autophagy and inhibiting apoptosis [70]. On the other hand, SIRT1 also represses autophagy in lung and cerebral Ischemia/Reperfusion (I/R) injury to suppress MEC apoptosis and inflammation [103,109]. Thus, SIRT1 appears to optimize autophagy in MECs under stress to protect against oxidative stress, inflammation, and apoptosis.

Recent evidence suggests a reciprocal regulatory relationship between SIRT1 and autophagy. In senescent cells, SIRT1 interacts more significantly with LC3, upregulating its cytosolic translocation and autophagic degradation [110]. Inhibition of autophagy promotes SIRT1 expression and induces proliferation of endothelial progenitor cells [111]. Similarly, in Parkinson’s disease (PD) mouse models, under oxidative-stress-induced by MPP+, a well-known parkinsonogenic agent, all human sirtuins were identified as autophagy substrates, demonstrating accelerated autophagy-dependent degradation [112]. However, sulfhydration-induced SIRT1 activation upregulated autophagy markers, which attenuated MPP+ induced neuronal damage [113], supporting that autophagic SIRT1 depletion induces senescence, whereas SIRT1-induced autophagy confers cytoprotection. However, SIRT1-induced autophagy may also be detrimental. Zhan et al. found that pulmonary hypoxia/reoxygenation injury leads to miR-141-3p inhibition, which induces SIRT1 activation. Conversely, SIRT1 deacetylates and stabilizes beclin-1 to enhance autophagy, which exacerbates pulmonary microvascular endothelial cell (PMEC) injury [114].

#### 3.2.3. SIRT1-PARP Has a Bidirectional Interaction in Regulating Microvascular Function

In response to DNA damage, poly (ADP-ribose) polymerase 1 (PARP1) consumes NAD+ to upregulate DNA repair mechanisms. In chronic inflammation, this leads to the depletion of NAD+ and is a major mechanism causing the downregulation of SIRT1 activity [115,116]. Deletion of PARP1 increases NAD+ and SIRT1 activity, thereby promoting mitochondrial function [116]. HG conditions in DM activate PARP, which leads to inhibition of SIRT activity and expression [117]. The loss of SIRT1 due to HG-induced PARP1 activation is a crucial mechanism regulating NF-κB-MMP9-mediated mitochondrial dysfunction in diabetic retinopathy [104]. Conversely, by preserving mitochondrial function and inhibiting mtROS formation, SIRT1 also indirectly suppresses PARP activation in RMECs, accompanied by downregulation of NF-κB activity [118].

#### 3.2.4. SIRT1 Regulates Oxidative Stress and Mitochondrial Dysfunction

Senescent MECs exhibit pro-oxidative phenotypes. Impairment of mitochondrial function is a manifestation of chronic inflammation and oxidative stress, resulting in the production of mtROS and mitochondrial DNA fragments (mtDNA) [39], which induce inflammation and contribute significantly to oxidative stress. SIRT1 is crucial to mitochondrial biogenesis due to its direct interaction with the transcriptional coactivator PGC-1α [119], which stimulates proteins involved in oxidative phosphorylation, mtDNA replication, mitochondrial fusion/fission, and mitophagy. In addition, PGC-1α co-activates transcription factors that are crucial to mitochondrial antioxidative capacity [120]. In BMECs, induction of SIRT1 nuclear translocation by erythropoietin maintains endothelial vascular integrity and prevents apoptosis by regulating mitochondrial function during oxidative stress [121].

Nrf2 is a cytoprotective transcription factor that promotes the expression of major antioxidant proteins SOD, catalase, HO-1, and NQO-1 by binding the antioxidant response element (ARE) in response to oxidative stress [122] and plays a key role in preserving mitochondrial function [123]. SIRT1 directly deacetylates Nrf2 and also promotes its expression [124,125]. In addition, SIRT1 represses the expression of KEAP1, which increases ubiquitination and degradation of Nrf2, and needs to be cleaved for the nuclear translocation of Nrf2 [126]. Additionally, PGC-1α may direct antioxidant gene expression by upregulating Nrf2 [127] and is required for the antioxidant effects of SIRT1. Knockdown of PGC-1α significantly reduces SIRT1-induced activation of the antioxidant proteins MnSOD, catalase, Prx5, TR2, and FOXO3a [128].

In human RMECs, HG-induced miR-221 and miR-34a activation downregulated SIRT1, leading to the loss of mitochondrial biogenesis factors including PGC-1α, Nrf1, Nrf2, and TFAM, mitochondrial antioxidants TrxR2 and SOD2, and premature senescence [42,129], while activation of SIRT1 could prevent HG-induced ROS elevation and apoptosis via activating PGC-1α [43]. SIRT1 also appears to play a significant role in the antioxidative and vasoprotective effects of dulaglutide against HG-induced senescence and ED [91]. Furthermore, activation of SIRT1 by FOXO6 inhibition-mediated CTRP3 upregulation induces Nrf2 signaling to reduce cardiac micro-endothelial barrier permeability and prevent MEC apoptosis [130]. In the cerebral microvascular endothelium, oxidative stress impairs ZO-1, a crucial component of endothelial tight junctions, leading to loss of endothelial integrity [66]. In addition, SIRT1-induced Nrf2 activation upregulated NOX4, HIF-1α, and VEGF expression following intestinal ischemia/reperfusion, leading to increased angiogenesis, cell viability, and migration of human PMECs [131]. A similar relationship between SIRT1 oxidative stress and angiogenesis has been observed in BMECs [77]. This suggests that the mitoprotective and antioxidative interaction between SIRT1 and the PGC-1α/Nrf2 pathway may also contribute to micro-endothelial barrier permeability and angiogenesis/capillarization. In ex vivo human MECs, SIRT1 is also shown to downregulate the expression of pro-oxidant and aging mitochondria proteins p66Shc and arginase II, which further contribute to the maintenance of mitochondrial function, preventing mtROS generation while promoting NO bioavailability and proteins associated with the mitochondrial respiratory chain [10].

Crucially, SIRT1 also regulates the destruction of dysfunctional mitochondria by promoting mitophagy in MECs. Activation of SIRT1 by adiponectin induces stability of PINK1, a molecular marker for abnormal mitochondrial membrane potential, which signals mitophagy of specific dysfunctional mitochondria to suppress PMEC oxidative damage, inflammation, and apoptosis, while knockdown of SIRT1 suppresses PINK1 expression and abolishes the antioxidative effects of mitophagy [44]. Similarly, upregulation of SIRT1/FOXO3a signaling also ameliorated OGD/R-induced cerebral MEC injury by upregulating mitophagy [67].

### 3.3. SIRT1 Plays a Dual Role in Micro-Neovascularization

The pro-angiogenic effects of SIRT1 have been well illustrated in many studies. However, recent evidence shows that SIRT1 also suppresses stress-induced pathological micro-angiogenesis, such as that observed in diabetic retinopathy.

#### 3.3.1. SIRT1 Promotes Angiogenesis by Inhibiting DLL4-Notch Signaling

SIRT1 promotes microvascular angiogenesis primarily via the inhibition of Notch signaling. Upon VEGFR activation, tip-cell Delta-like-ligand 4 (DLL4) binds to stalk-cell Notch, which reduces stalk-cell VEGF sensitivity and inhibits angiogenesis. Meanwhile, tip-cell Jagged 1 (Jag1) competes with DLL4 to promote angiogenesis [132,133]. In endothelial progenitor cells, upregulation of SIRT1 activity by NAMPT following mouse hindlimb ischemia leads to increased SIRT1-dependent deacetylation of the Notch intracellular domain (NICD), inhibiting the DLL4-Notch signaling pathway and upregulating VEGFR-2 and VEGFR-3 to promote angiogenesis. Furthermore, SIRT1 upregulation renders Notch more sensitive to pro-angiogenic Jagged1 as compared to anti-angiogenic DLL4. These changes enhance capillary density and improve post-ischemic blood flow recovery [134]. SIRT1 activation by H2S-induced NAD+ elevation led to interaction with NICD under hypoxic conditions, negatively regulating Notch signaling in micro-endothelial cells to promote sprouting and ameliorate peripheral nerve injury [17,135].

#### 3.3.2. SIRT1 Suppresses HIF-1α-Induced Pathological Angiogenesis

**Figure 2:**
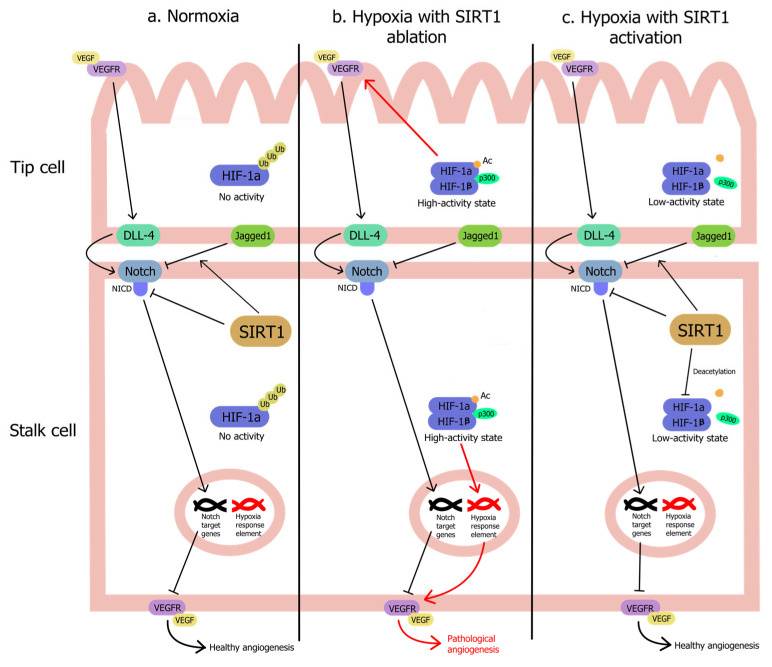
SIRT1 suppresses HIF-1α activity during hypoxia to prevent pathological angiogenesis. (**a**) Under normoxic conditions, hypoxia-inducible factor-1a (HIF-1α) is degraded via the ubiquitin-proteasome pathway, while Notch signaling is inhibited by SIRT1 via promoting Notch sensitivity to Jagged1 and deacetylating the Notch intracellular domain, which promotes healthy angiogenesis. (**b**) Under hypoxic conditions, acetylated HIF-1α leads to complete recruitment of p300 to the HIF-1α-HIF-1β complex, resulting in a high activity state on the hypoxia response element (HRE) and drastic upregulation of vascular endothelial growth factor (VEGF) and VEGF receptors (VEGFR) to induce pathogenic angiogenesis. (**c**) SIRT1 upregulation during hypoxia deacetylates HIF-1a, blocking the recruitment of p300 and reducing its activity on the HRE. In addition, SIRT1 promotes healthy angiogenesis by inhibiting Notch signaling.

SIRT1 closely interacts with HIF-1α, a master regulator of oxygen homeostasis involved in various pathologies, governing the activity of genes involved in angiogenesis, vascular remodeling, and energy metabolism by targeting VEGF, platelet-derived growth factor (PDGF), and van Willebrand factor (vWF) expression [136,137]. Under normoxia, HIF-1α is inactivated by cytoplasmic ubiquitin-proteasome-mediated degradation. However, under hypoxic conditions, SIRT1 activation stabilizes and accumulates HIF-1α in a low-activity state by blocking p300 recruitment. If SIRT1 is ablated during hypoxia, p300 fully binds to the HIF-1α-HIF-1β complex, leading to a high activity state of HIF-1α target genes [138]. Under hypoxic/HG conditions, linagliptin-induced activation of SIRT1 stabilizes HIF-1α, leading to its accumulation to reduce abnormal proliferation and migration of rat BMECs [92]. Ablation of endothelial SIRT1 under transverse aortic constriction increases protein expression of HIF-1α [55]. Hypoxic SIRT1 activation promotes HIF-1α stability and accumulation in a low-activity state, resulting in the suppression of pathological angiogenesis (Figure 2).

In diabetic microangiopathy, endothelial SIRT1 dysfunction causes capillary loss via enhanced Notch signaling, including upregulated DLL4, Notch target genes Hey1, Hes1, and Notch intracellular domain (NICD) in peritubular and RMECs [32,139], along with diminished NO production [33]. SIRT1 upregulation by NAD+ restored myocardial capillary density and ameliorated myocardial fibrosis [35,56]. HG-exposed MECs overexpress miR-34a, inhibiting SIRT1 and impairing angiogenesis, whereas metformin inhibits miR-34a to activate SIRT1, reversing these effects [140]. In addition, SIRT1 promotes healthy endothelial tube formation and neovascularization by regulating pro-angiogenic chemokine secretion in early outgrowth cells [141]. Simultaneously, a pseudo-hypoxic state induced by diabetic metabolic changes diminishes NAD+ and SIRT1 function, whereas loss of SIRT1 causes HIF-1α activation, resulting in pathological angiogenesis and kidney fibrosis [34].

In diabetic retinopathy, the role of pathological angiogenesis is well characterized. Hypoxic retinal microvascular conditions lead to HIF-1α activation-induced VEGF overproduction, which is associated with abnormal angiogenesis, increased capillary permeability, and retinal dysfunction [36]. Overexpression of SIRT1 suppresses HIF-1α-induced VEGF overproduction [142], resulting in reduced diabetic retinal angiogenesis [27,38], whereas SIRT1 inhibition promotes pathological angiogenesis via HIF-1α activation [37]. This is further supported by the finding that in nondiabetic oxygen-induced-retinopathy, wherein HIF-1α is inactive, deletion of SIRT1 suppresses angiogenesis and retinal revascularization, with a significant downregulation of VEGF-A/VEGFR-2 [143]. As such, SIRT1 seems to promote healthy micro-angiogenesis while inhibiting pathological HIF-1α-induced diabetic angiogenesis.

#### 3.3.3. SIRT1-eNOS Axis Promotes Tissue Capillarization

SIRT1-eNOS also contributes to the maintenance of tissue capillarization. In the kidney, GDNF-modified human adipose mesenchymal stem cell-exosome-induced SIRT1 signaling is associated with increased phosphorylated eNOS, which is positively correlated with peritubular capillary quantity in vivo [33]. In endothelial colony-forming cells, RSV administration increased eNOS expression, NO production, proliferation, and capillary-like outgrowth sprout formation [144].

### 3.4. SIRT1 Upregulates Tight Junction Proteins to Maintain Micro-Endothelial Cell-Cell Junctions

Endothelial tight junctions (TJs) are composed of transmembrane and scaffolding proteins, including claudin-5, occludin, and TJ proteins 1 and 2 (ZO-1 and ZO-2). Dysregulation or downregulation of TJ proteins increases endothelial permeability and extravasation of toxins [145]. In particular, TJs are crucial for maintaining the BBB, which prevents neuronal damage by toxins, immune factors, and plasma proteins [146]. Aging, cerebral ischemia, and OGD/R have been shown to reduce the expression of SIRT1, occludin, claudin-5, VE-cadherin, and ZO-1 in BMECs, leading to dysregulated BBB permeability [59,109]. Through regulating the expression and complex organization of TJ proteins, SIRT1 is critical to maintaining BBB function. Overexpression of SIRT1 in BMECs rescued claudin-5, occludin, ZO-1, and VE-cadherin expression, while stabilizing claudin-5/ZO-1 interactions to protect against senescence-induced brain endothelial barrier hyperpermeability, cerebral ischemia, and OGD/R [59,63,69]. Specifically, RSV treatment increased claudin-5 expression and decreased inflammation following TNFα treatment [147]. Mechanistically, inhibition of RhoA/ROCK signaling by SIRT1 may increase endothelial barrier integrity. In pulmonary MECs, SIRT1 inhibits RhoA/ROCK signaling to increase occludin, claudin-5, ZO-1, and ZO-2 expression during LPS-induced inflammation [148]. A similar effect has also been observed in the brain, wherein catalpol-induced RhoA/ROCK inhibition was associated with increased TJ protein expression and reduced BBB permeability [149], which may further explain the neuroprotective activity of RhoA/ROCK inhibition against amyloidogenesis [150].

### 3.5. SIRT1 and Microcirculatory Thrombosis

ED has also been associated with hyperinflammation. Inflammatory factors released by cells upon stress, cell death, or immune cells contribute significantly to thrombosis. The association between thrombosis and inflammation is referred to as thromboinflammation. Blood flow impairment due to microcirculatory thromboinflammation is a major pathological factor in aging-related diseases. The mitoprotective, anti-inflammatory, and antioxidant properties of SIRT1 make it a potential target for preventing microvascular thromboinflammation.

#### 3.5.1. SIRT1 Regulates Endothelial Glycocalyx Function

SIRT1 maintains the endothelial glycocalyx, which plays a crucial role in microcirculatory thrombosis, and prevents its shedding in response to inflammatory stimuli [107]. The endothelial glycocalyx plays a mechanosensory role in the microvascular endothelium to stimulate NO and prostacyclin production [151], and the loss of endothelial glycocalyx is associated with greater platelet adhesion to the endothelial wall [152]. Similarly, upregulation of NO production has been shown to inhibit endothelial adhesion molecule expression, P-selectin, vWF, and platelet activation [151], and endothelial deficiencies in eNOS further exacerbate microvascular thrombosis [153].

#### 3.5.2. SIRT1 Interacts with Prostacyclin Signaling

SIRT1 interacts with prostacyclin to modulate microvascular thrombosis. Released in response to shear stress, prostacyclin acts similarly to NO to produce a vasodilatory response. Prostacyclin is produced by COX-1 and COX-2. COX-1 also produces pro-thrombotic Thomboxane A2 (TXA2), while COX-2 produces proinflammatory Prostaglandin E2 [154]. It is thought that thromboprotection by low-dose aspirin is derived from shifting the balance between PGI and TXA2 in favor of PGI production [155]. This is supported by the fact that a well-known side effect of selective COX-2 inhibition, the dominant producer of prostacyclin [156], is the increased risk of thrombotic events [157], which have been largely attributed to the inhibition of prostacyclin synthesis [158]. Prostacyclin is positively associated with SIRT1, as antagonism of prostacyclin receptors significantly reduced SIRT1 levels, and SIRT1 inhibition increased the expression of tissue factor, the primary initiator of coagulation, and promoted occlusive thrombi generation in mice [159]; whereas administration of exogenous NAD+ in the form of 1-methylnicotinamide inhibited platelet-mediated thrombosis via prostacyclin activation [160].

#### 3.5.3. SIRT1 Directly Regulates Platelet Activity and Lifespan

SIRT1 is expressed in human platelets, and platelet-specific SIRT1 inhibition causes apoptotic changes and thrombocytopenia due to increased acetylated p53 [161]. However, while SIRT1 is necessary for platelet function, SIRT1 activation/overexpression inhibits the expression of platelet-activating factor receptors and reduces platelet aggregation, attenuating thrombus formation [162]. SIRT1 activation also reduces fibrinogen binding α-granule release and contributes to platelet cytoskeletal reorganization to reduce aggregation [163]. In addition, SIRT1 increases platelet phagocytosis by human endometrial MECs to delay senescence via the deacetylation of Akt [164], showing that SIRT1 also plays a role in regulating platelet lifespan. This is supported by clinical trial evidence, which shows that SIRT1 activation by SRT2104 lowers plasma levels of prothrombin, IL-6, and IL-8 in healthy subjects [165].

## 4. Targeting Microvascular SIRT1 in Aging-Related Disease

Many substances have been found to modulate SIRT1, including plant derivatives, nucleic acids, and clinically proven drugs. This section discusses microvascular SIRT1 modulators that are currently under development.

### 4.1. Natural Modulators of SIRT1

#### 4.1.1. Resveratrol

RSV, a natural compound found mostly in red grapes, is the most well-known SIRT1 activator and is often treated synonymously with SIRT1 activation. Although RSV seems to provide clear benefits to endothelial function in animals, its effect in human trials has shown inconsistent results, as illustrated in a recent systematic review and meta-analysis [166]. This may be due to its low oral bioavailability [167]. To date, no optimal dose has been established for humans. Many targeted delivery methods have been developed, including lipid nanoparticles [168], cyclodextrin complexes, and nanosponges [169]. Clinical trials have shown that RSV elicits very few adverse reactions with no serious adverse events despite doses of up to 5 g per day. Given this, larger trials of sufficient duration with multi-morbid study populations are needed to clearly elucidate the effects of RSV in humans [166].

Despite its widespread use as a SIRT1 activator, RSV also possesses off-target effects, which may be SIRT1-independent. For instance, carcinogen-induced BMEC inflammation led to the elevation of MMP-9 and COX-2, which was reversible by RSV; however, silencing of SIRT1 did not influence the effects of RSV on MMP-9 and COX-2 [170]. The effects of RSV on SIRT1 activation may also be attenuated in diabetic conditions. In LPS-induced neuroinflammation, micro-endothelial cells subjected to a hyperglycemic state demonstrated reduced SIRT1 expression and attenuated increase in SIRT1 activity following RSV administration compared to normoglycemic and hypoglycemic conditions [171], suggesting that diabetic individuals may require higher doses for therapeutic effect.

#### 4.1.2. Other Natural Modulators

Various organic SIRT1 modulators have been shown to protect against streptozotocin-induced diabetic retinopathy models. Coumestrol, an estrogenic phytochemical, increasesSIRT1 expression, suppressing inflammation, oxidative stress, and apoptosis in human RMECs [172]. Wogonoside, a flavonoid, activates SIRT1 and suppresses abnormal angiogenesis, permeability, proliferation, and migration of RMECs [173]. Stachydrine, a plant metabolite, activated AMPK/SIRT1 signaling to upregulate RMEC autophagy alongside suppressing ROS and inflammation [46].

Organic SIRT1 modulators also ameliorated brain injury in middle cerebral artery occlusion/reperfusion (MCAO/R) and OGD/R models of cerebral ischemia. Safranal, the major volatile component of saffron, upregulates SIRT1 expression to promote survival, proliferation, and angiogenesis in rat BMECs following MCAO/R and OGD/R [174]. 14,15-epoxyeicosatrienoic acid, an arachidonic acid metabolite, protected against OGD/R-induced BMEC injury by inducing SIRT1/FOXO3a-mediated mitophagy [67]. Hydroxysafflor Yellow A, a safflower derivative used in Traditional Chinese Medicine (TCM), increased SIRT1 expression, protected BBB integrity, induced angiogenesis, and promoted survival of BMECs following OGD/R [66,175]. Ligustrazine, found in fermented cocoa and natto beans, promotes SIRT1 expression and angiogenesis in BMECs following MCAO/R [176]. In addition, by inhibiting miR-34a-5p to activate SIRT1, ligustrazine is also shown to prevent CMD, platelet activation, and inflammation to alleviate coronary microembolization [54].

Organic SIRT1 modulators also influence physiological regulation by SIRT1 in areas such as lipid metabolism and skeletal muscle function. Boysenberry polyphenols are shown to significantly upregulate endothelial SIRT1, leading to enhanced capillarization and function of BAT, which plays a role in increasing systemic glucose tolerance and thermogenesis [22]. Lycopene, a carotenoid pigment, activated SIRT1, increasing skeletal muscle capillary density and preventing microvascular endothelial damage in aging rats [13]. Through promoting SIRT1 activation, salvianolic acid B, a compound derived from the TCM herb Salvia miltiorrhiza, promoted anti-inflammatory M2 macrophage polarization and angiogenesis, alongside increased muscle capillary density and blood perfusion following mouse limb ischemia [177] (Table 1).

### 4.2. Endogenous SIRT1 Modulators

#### 4.2.1. Nicotinamide Adenine Dinucleotide (NAD+) Modulators

Functionally, the activity of SIRT1 is regulated most significantly by the NAD+/NADH ratio. Thus, boosting cellular NAD+ is a prominent pharmacological strategy for activating SIRT1 [178]. Administration of NAD+ precursors, such as nicotinamide riboside (NR) and NMN, is a popular approach. NMN treatment promotes angiogenesis and suppresses ROS production in CMECs, a process that can be reversed by pharmacological inhibition of SIRT1 via EX-527 [77]. Treatment of mice with NMN also increases exercise-induced skeletal muscle capillary density in elderly mice via SIRT1 activation [17]. Meanwhile, NR has been shown to significantly alleviate intestinal MEC dysfunction by activating the SIRT1-eNOS pathway and reducing ROS production under inflammatory conditions such as TNFα stress and necrotizing enterocolitis [89].

Targeting nicotinamide phosphoribosyl transferase (NAMPT) is another approach to increase biosynthesis of NAD+. FK866 inhibited NAMPT and impaired mobilization of EPCs from the bone marrow upon ischemic stress, reducing angiogenesis and vascular repair, whereas NAMPT overexpression induced opposite effects through SIRT1-dependent enhancement of NICD deacetylation. While silencing SIRT1 reversed the beneficial effects of NAMPT overexpression [134]. Similarly, targeting the nicotinamide mononucleotide adenylyl transferase (NMNAT) pathway for the synthesis and salvage of NAD+ is viable. Intranasal administration of recombinant human NMNAT1 in mice leads to enhanced BBB integrity by upregulating SIRT1 [63].

Besides directly intervening in the NAD+ pathway, studies have also proposed indirect agonism of NAD+-SIRT1 signaling. On its own, H2S induces post-translational sulfhydration of SIRT1, which upregulates its deacetylase activity [179]. The promotion of endogenous H2S production via S-propargyl-cysteine leads to SIRT1 upregulation and microvascular reconstruction following peripheral nerve injury [135]. A combination of sodium hydrosulfide (NaSH), an H2S donor, and NMN supplementation synergistically activates SIRT1 to further promote exercise-induced capillarization of skeletal muscles [17]. Co-administration of H2S boosters and NMN could represent an axis for future research on managing aging-associated frailty (Table 2).

#### 4.2.2. Hormones and Hormone-like Substances

Various hormones and hormone-like substances have also been studied as SIRT1 modulators. Melatonin is a well-known SIRT1 activator [180,181]. In coronary MEC of streptozotocin-induced diabetic mice, melatonin activated AMPK/SIRT1 signaling to reduce oxidative stress and increase antioxidant capacity [41]. Phoenixin 20, a bioactive peptide with hormone-like actions to regulate hypothalamo−pituitary−gonadal hormones and reproduction [182], has been shown to activate SIRT1, leading to inhibition of NLRP3-mediated inflammation and oxidative stress [183]. Desacyl ghrelin, the precursor of ghrelin, protects RMECs from oxidative-stress-induced apoptosis via SIRT1-mediated upregulation of antioxidant enzymes [184].

#### 4.2.3. Non-Coding RNA (ncRNA) Modulators of SIRT1

##### MicroRNA (miR)

Post-transcriptionally, degradation of SIRT1 mRNA is generally induced by ncRNAs known as microRNAs (miRs). As such, miRs have been a major focus of SIRT1 research in recent years. MiR-195 has been extensively studied in microcirculation. Under diabetic conditions, miR-195 is upregulated, leading to the loss of SIRT1 expression in RMECs, increased apoptosis, and reduced proliferation of RMECs, which could be reversed upon SIRT1 overexpression or administration of an miR-195 antagonist (anti-miR) [185]. Similarly, in cardiac MECs, miR-195 expression is increased in response to streptozotocin-induced diabetes, which is associated with reduced SIRT1 levels, impaired myocardial function, oxidative stress, and myocardial hypertrophy, whereas silencing of miR-195 reverses these effects [35]. Various other miRs targeting SIRT1 have been investigated in the microcirculation, with diabetic retinopathy being a major focus. Under HG conditions, miR-221, miR-377, miR-34a, miR-30b, and miR-29b-3p are upregulated, leading to SIRT1 downregulation, pathological proliferation, migration, inflammation, apoptosis, senescence, oxidative stress, and angiogenesis in RMECs, contributing to the pathogenesis of diabetic retinopathy [27,38,42,129,186]. In the heart, miR-34a-5p similarly suppressed SIRT1 and induced platelet activation, inflammation, and CMD, which was reversible by ligustrazine through inhibiting miR-34a-5p to upregulate SIRT1 [54]. Similarly, in a pulmonary I/R model, miR-145 inhibited SIRT1 to induce NF-κB-mediated inflammation, autophagy, and lung injury [103]. Therefore, the development of anti-miRs and miR-inhibiting compounds to promote SIRT1 activity presents a clear direction for research on aging-related diseases. Delivery methods targeting microcirculatory miRs are also being developed. For example, delivery of a locked nucleic acid-based miR-92a inhibitor via deoxycholic acid-modified polyethyleneimine polymeric conjugates as polyplex nanoparticles within a hydrogel reservoir was able to induce SIRT1 expression and localized angiogenesis in mice subcutaneous tissue and elevate capillary density in a chicken chorioallantoic membrane model [187] (Table 3).

However, miR upregulation could also elicit beneficial interactions with SIRT1. For example, miR-141-3p rescues lung injury by inhibiting SIRT1-induced PMEC autophagy [114]. Regular exercise induces both SIRT1 and miR-29 expression [18] to alleviate ED. Although miR-29 does inhibit SIRT1 [188], its plasma levels were found to be correlated with increased antioxidant activities [18]. Furthermore, certain miRs promote SIRT1 signaling. For example, miR-16-5p inhibition leads to downregulation of SIRT1 in BMECs, aggravating cerebral infarction [65]. Similarly, miR-126 overexpression promotes SIRT1/Nrf2 signaling, ameliorating OGD/R-induced injury in human umbilical vein endothelial cells [189]. Therefore, the potential benefits of miR-SIRT1 interaction should not be neglected.

##### Circular RNA (circRNA)

Circular RNA is a single-stranded, covalently closed RNA that acts as a transcriptional regulator, miR sponge, and protein template [190]. In the microvasculature, circ-HIPK3 sponges miR-148b-3p, downregulating cyclin-dependent kinase 5 (CDK5) and CDK5 receptor 1 expression, which ultimately upregulates SIRT1 to promote post-stroke BMEC survival and mitochondrial function [64]. Similarly, circ-cPWWP2a sponges miR-579 in diabetic microvascular pericytes to upregulate SIRT1 expression, which correlates with improved retinal function [191].

Macrovascular evidence suggests that circ-SIRT1, derived from the circularization of exons 2 and 7 of the SIRT1 gene, is crucial to inflammation, hypertension, and atherosclerosis, and is significantly downregulated during aging, neointima formation, VSMC injury, pulmonary arterial hypertension, and angiotensin-II treatment [192,193,194,195]. Circ-SIRT1 delivery sponges miR-3681-3p/5195-3p and miR-132/212 to stabilize SIRT1 protein from degradation and enhance SIRT1 expression, inhibiting cardiac hypertrophy and ameliorating VSMC inflammation via SIRT1/NF-κB signaling [192,193]. Furthermore, circ-SIRT1 inhibits VSMC proliferation by suppressing the c-Myc/cyclin-B1 axis, while also preventing p53-induced VSMC senescence [194,195]. While the effects of circ-SIRT1 have yet to be demonstrated in the microvasculature, its anti-inflammatory and anti-hypertensive effects, alongside its ability to suppress miR-induced SIRT1 inhibition, could also play an important role in aging-related disease.

##### Long Non-Coding RNA

LncRNAs are a multifunctional class of RNAs generally defined as non-translated transcripts >200 nucleotides in length [196]. The interaction between lncRNAs and microcirculatory SIRT1 activity has been the most studied in the brain. Cerebral infarction or ischemia has been shown to elevate lncRNA KCNQ1OT1, MALAT1, NEAT1, and Snhg12, while suppressing Snhg8 [62,65,70,71,197]. LncRNAs have been shown to target miRs associated with SIRT1 function to influence the microvasculature. LncRNAs Snhg8 and Snhg12 sponge miR-425-5p and miR-199a, respectively, and upregulate SIRT1 to promote BBB integrity and AMPK activation, while suppressing post-ischemic NF-κB-mediated inflammation in BMECs [62,197]. Similarly, lncRNA MALAT1 promotes SIRT1-mediated autophagy by sponging miR-200c-3p to protect BMECs against OGD/R, and lncRNA NEAT1 sponges miR-377 to upregulate SIRT1, which is associated with angiogenesis and survival of BMECs following OGD/R [70,71]. However, lncRNAs can also sponge SIRT1-activating miRs. For instance, lncRNA KCNQ1OT1 aggravates cerebral infarction by sponging miR-16-5p, leading to polypyrimidine tract binding protein 1 activation and subsequent SIRT1 downregulation in BMECs associated with inflammation and diminished angiogenesis [65].

##### tRNA-Derived Stress-Induced RNA (tiRNAs)

TiRNAs are a novel type of ncRNA generated by specific cleavage of tRNAs into 30-40 codon-long sequences [198]. Few studies have investigated the effects of tiRNAs on SIRT1 expression. However, it seems that tiRNAs may significantly regulate SIRT1. tiRNA-Val, derived from mature tRNA-Val, is upregulated in human RMECs under HG conditions, decreasing SIRT1 expression, and ensuing HIF-1α upregulation enhanced RMEC proliferation [37], suggesting a role for tiRNA-Val in diabetic retinopathy-associated pathological angiogenesis.

### 4.3. Synthetic SIRT1 Modulators

#### 4.3.1. SRT1720

SRT1720 has been studied as a direct SIRT1 activator in microcirculation, ameliorating obesity-induced ED by promoting antioxidant protein expression while downregulating mitochondrial-aging proteins [10]. SRT1720 also increases PMEC TJ permeability by inhibiting the RhoA/ROCK signaling pathway [148]. In diabetic ulcers, SRT1720 enhanced survival, proliferation, and migration of embryonic artery cluster of differentiation 133+ cells seeded within the wound dressing, which accelerated healing of diabetic ulcers in mice by enhancing wound angiogenesis and capillarization [199].

#### 4.3.2. SIRT1 Inhibitors

While most studies have demonstrated that SIRT1 activation exerts microvascular vasoprotection, SIRT1 inhibition may also be beneficial. For instance, SIRT1 inhibition can elicit anti-inflammatory effects. EX-527 is a popular choice for testing the reversibility of the effects of purported SIRT1 activators. However, during the hypo-inflammatory phase of sepsis, SIRT1 inhibition by EX-527 represses adhesion molecule expression in MECs while stabilizing blood pressure and microvascular perfusion [200]. Similarly, inhibition by sirtinol diminished human dermal MEC inflammation by suppressing adhesion molecule expression and chemokine signaling in TNFα, or IL-1β stimulated cells [201]. In addition, SIRT1 inhibition by salermide increased post-ischemic BBB integrity, whereas SIRT1 overexpression had the opposite effect. Suppression of SIRT1 by salermide also attenuated mtROS generation, further preventing post-ischemic BBB permeability and cell damage. In this study, SIRT3, but not SIRT1, protected BBB integrity, and its knockdown significantly increased BBB permeability [60]. Despite this, the mechanisms and circumstances wherein SIRT1 inhibition confers microcirculatory protection are poorly understood and require further investigation.

#### 4.3.3. Other SIRT1 Modulators

Several clinically proven drugs have been shown to function as SIRT1 modulators. In diabetic microangiopathy, cilostazol promotes retinal angiogenesis and upregulates SIRT1 via the adiponectin/adipoR signaling pathway, which is necessary for SIRT1 expression [90]. Fenofibrate, a PPARa agonist, increases SIRT1 activity and reduces NF-κB activation to suppress retinal inflammation [202]. Methylene blue, used in the management of methemoglobinemia, protects RMECs against inflammation, apoptosis, and oxidative stress by increasing SIRT1 expression [26].

In the brain, dihydroartemisinin, an antimalarial, ameliorated MCAO/R-induced cerebral injury by upregulating autophagy via SIRT1/FOXO1 signaling [109]. Similarly, donepezil ameliorates BMEC dysfunction following OGD/R by activating SIRT1/FOXO3a/NFkB signaling to promote migration angiogenesis and reduce BBB permeability [69]. Furthermore, SIRT1 activation by cystatin C could shift APP processing toward a non-amyloidogenic pathway in BMECs, providing a potential target for the treatment of Alzheimer’s disease [79].

Evidence suggests that the microcirculatory effects of SIRT1 are heavily involved in the pharmacological mechanism of various antidiabetics, such as gliquidone, metformin, GLP-1 agonists, and DPP-4 inhibitors. Expression of SIRT1 is significantly increased by metformin in MECs under HG conditions, which modulates downstream SIRT1 pathways to alleviate HG-induced senescence, apoptosis, oxidative stress, and inflammation [118,203]. In diabetic retinopathy, gliquidone regulated Notch-mediated angiogenesis to alleviate retinal injury via SIRT1 [139]. Similarly, SIRT1 is involved in the neurological and retinal protective effects of DPP-4 inhibitors and GLP-1 agonists. GLP-1 activation increases SIRT1 to ameliorate HG-induced RMEC senescence, inflammation, and angiogenesis and improves eNOS function. Specifically, SIRT1 activation by dulaglutide contributes to genome stability by restoring telomerase activity [91,204]. On the other hand, research on DPP-4 inhibitors also demonstrated its neuroprotective effects via SIRT1 activation in diabetes, improving angiogenesis, mitochondrial function, and NO bioavailability while suppressing oxidative stress, abnormal proliferation, and migration of BMECs under HG, ischemia, and even psychological stress [80,92,205].

Hydrogen-rich saline has been shown to elicit anti-inflammatory effects by upregulating the SIRT1/NF-κB signaling pathway to prevent microvascular endothelial glycocalyx shedding and maintain micro-endothelial function in sepsis-induced kidney injury [105]. Similarly, Tubastatin A, a histone deacetylase 6 inhibitor, has been shown to increase SIRT1 expression and alleviate oxidative stress in DR [206]. Many compounds and existing drugs are emerging as SIRT1 modulators, and further studies on their mechanisms may reveal great insights into the link between microvascular aging and disease.

## 5. Conclusions

This review highlights the integral role of SIRT1 pathways in microcirculation, including suppression of NF-κB-mediated inflammation, thrombosis, and oxidative stress, while promoting mitochondrial function via PGC-1α/Nrf2-mediated antioxidant signaling. These actions collectively enhance microvascular endothelial function and prevent premature senescence.

Notably, in the microcirculation, SIRT1 exhibits a unique bidirectional role in the regulation of angiogenesis. It suppresses HIF-1α-induced microvascular angiogenesis associated with diabetic microangiopathy while promoting physiological capillarization of skeletal muscle and adipose tissue. SIRT1 also plays a crucial role in maintaining micro-endothelial barrier function by upregulating the expression of TJ proteins. SIRT1 modulates the interactions between TJ proteins, such as stabilizing the claudin-5/ZO-1 complex. Further research is required to understand the underlying mechanisms of such interactions.

SIRT1 is essential for the maintenance of microvascular EG, prostacyclin signaling, and platelet function to prevent microvascular thrombosis, although the specific mechanisms underlying EG regulation by SIRT1 remain to be elucidated.

The present review also discusses the effects of various SIRT1 modulators on microcirculation. Although there are an abundance of animal studies supporting the microvascular function of SIRT1, there is a lack of clinical evidence for the microcirculation-specific effects of SIRT1. A wide range of novel SIRT1 modulators are being developed alongside drug delivery methods to tackle the bioavailability issues of SIRT1 activators, such as RSV.

As the focus of medicine in aging-related disease shifts toward the microvasculature, SIRT1 presents great potential as a treatment target for improving microvascular function. However, larger trials of sufficient duration with more diverse study populations are required to clearly elucidate the effects of SIRT1 in human microcirculation.

## Figures and Tables

**Table 1:** Natural SIRT1 activators and their mechanisms.

Natural SIRT1 Intervention	Primary Mechanisms	Reference
Boysenberry polyphenols	Enhanced capillarization and BAT function, increased systemic glucose tolerance, and optimized thermogenesis.	[13]
Lycopene	Increase skeletal muscle capillary density, prevent MEC damage	[22]
Stachydrine	Inducing RMEC autophagy, suppressing ROS and inflammation,	[46]
Ligustrazine	Promote angiogenesis of BMECs while suppressing CMD, platelet activation, inflammation, and coronary micro-embolization	[54,176]
14,15 epoxyeicosatreinoic acid	Promote mitophagy	[67]
Hydroxysafflor Yellow A	Promote BBB integrity, angiogenesis, and survival of BMECs	[66,175]
Coumestrol	Suppressing inflammation, oxidative stress, and apoptosis in human RMECs	[172]
Wogonoside	Suppressing abnormal angiogenesis, permeability, proliferation, and migration of RMECs	[173]
Safranal	Promote survival, proliferation, and angiogenesis in BMECs	[174]
Salvianolic acid B	Promote anti-inflammatory M2 macrophage polarization, angiogenesis, muscle capillary density, and blood perfusion.	[177]

**Table 2:** NAD+ modulators targeting SIRT1 activation and their mechanisms.

NAD+ Modulating Intervention	Primary Mechanisms	Reference
Sodium hydrosulfide+ NMN	Synergistic SIRT1 activation, promoting exercise-induced capillarization of skeletal muscle	[17]
Nicotinamide mononucleotide (NMN)	Promote angiogenesis and suppress ROS production in CMECs, and increase exercise-induced skeletal muscle capillary density	[17,77]
Recombinant human nicotinamide mononucleotide adenylyl transferase	Enhanced BBB integrity	[63]
Nicotinamide riboside	Prevent intestinal MEC dysfunction and reduce ROS production under inflammatory conditions	[89]
S-propargyl-cysteine	Promote endogenous H2S production, upregulating SIRT1 and microvascular reconstruction following peripheral nerve injury	[135]

**Table 3:** MicroRNA and their effects on SIRT1 and microcirculation.

MicroRNA (miR)	Effect on SIRT1 and Microcirculation	Reference
miR-29	Inhibits SIRT1, but is induced by regular exercise and associated with increased antioxidant activity and reduced endothelial dysfunction.	[18,188]
miR-377	Inhibit SIRT1 expression aggravates cell cycle transition, angiogenesis, migration, and inflammation in human RMECs under HG conditions.	[27]
miR-195	Increased under diabetic condition. In RMECs, inhibit SIRT1 expression, increasing apoptosis and reducing proliferation. In CMECs, reduced SIRT1 impairs myocardial function, causing oxidative stress and myocardial hypertrophy	[35,185]
miR-30b	Negatively regulate SIRT1, promoting pathological angiogenesis in proliferative diabetic retinopathy.	[38]
miR-221	Inhibit SIRT1/Nrf2 signaling in human RMECs, promoting apoptosis under HG conditions.	[42]
miR-34a-5p	Suppress SIRT1 in CMECs induces platelet activation, inflammation, and CMD.	[54]
miR-16-5p	Inhibition of miR-16-5p downregulates SIRT1, exacerbating cerebral infarction in mice.	[65]
miR-145	Inhibit SIRT1, inducing NF-kB mediated inflammation, autophagy, and lung injury in a pulmonary I/R model.	[103]
miR-141-3p	Ameliorated lung injury by inhibit SIRT1-induced pulmonary MEC beclin-1-dependent autophagy in mice pulmonary H/R model.	[114]
miR-34a	Decrease SIRT1 levels, diminish mitochondrial function antioxidant capacity, and induce senescence in human RMECs under HG conditions.	[129]
miR-29b-3p	Downregulate SIRT1, decrease human RMEC viability, and upregulate apoptosis under HG conditions.	[186]
miR-92a	Inhibition of miR-92a induced SIRT1 expression and induced angiogenesis in subcutaneous tissue, elevating capillary density in a chicken chorioallantoic membrane model.	[187]
miR-126	Promote SIRT1/Nrf2 signaling, and attenuate oxidative/inflammatory response to OGD/R injury in HUVECs.	[189]
